# Resonance-Based Time-Frequency Manifold for Feature Extraction of Ship-Radiated Noise

**DOI:** 10.3390/s18040936

**Published:** 2018-03-22

**Authors:** Jiaquan Yan, Haixin Sun, Hailan Chen, Naveed Ur Rehman Junejo, En Cheng

**Affiliations:** 1 Key Laboratory of Underwater Acoustic Communication and Marine Information Technology, Ministry of Education, Xiamen University, Xiamen 361005, China; jquan.yan@gmail.com (J.Y.); hxsun@xmu.edu.cn (H.S.); hailan.chen@163.com (H.C.); naveedrehmanjunejo@stu.xmu.edu.cn (N.U.R.J.); 2 School of Information Science and Engineering, Xiamen University, Xiamen 361005, China; 3 School of Science, Jimei University, Xiamen 361021, China

**Keywords:** ship-radiated noise, resonance-based sparse signal decomposition, manifold learning, phase space reconstruction, resonance-based time-frequency manifold

## Abstract

In this paper, a novel time-frequency signature using resonance-based sparse signal decomposition (RSSD), phase space reconstruction (PSR), time-frequency distribution (TFD) and manifold learning is proposed for feature extraction of ship-radiated noise, which is called resonance-based time-frequency manifold (RTFM). This is suitable for analyzing signals with oscillatory, non-stationary and non-linear characteristics in a situation of serious noise pollution. Unlike the traditional methods which are sensitive to noise and just consider one side of oscillatory, non-stationary and non-linear characteristics, the proposed RTFM can provide the intact feature signature of all these characteristics in the form of a time-frequency signature by the following steps: first, RSSD is employed on the raw signal to extract the high-oscillatory component and abandon the low-oscillatory component. Second, PSR is performed on the high-oscillatory component to map the one-dimensional signal to the high-dimensional phase space. Third, TFD is employed to reveal non-stationary information in the phase space. Finally, manifold learning is applied to the TFDs to fetch the intrinsic non-linear manifold. A proportional addition of the top two RTFMs is adopted to produce the improved RTFM signature. All of the case studies are validated on real audio recordings of ship-radiated noise. Case studies of ship-radiated noise on different datasets and various degrees of noise pollution manifest the effectiveness and robustness of the proposed method.

## 1. Introduction

Underwater ship-radiated noise, in which entire spectra are widely distributed from as low as 5 Hz to as high as 100 KHz, contributes dramatically to oceanic ambient noise [1]. Ship-radiated noise is composed of four types due to the generated sources: propulsion noises, propeller noises, auxiliary noises and hydrodynamic noises [2]. It is known that the broadband and tonal components are caused by the propeller and associated cavitation noises [1,3]. Quasi-periodic harmonics with low-frequency narrowband components are produced by the propulsion engines and propellers, whose amplitudes and frequencies are independent of ship speed [4,5,6]. Therefore, the harmonic elements play an important role in the detection and classification of ship-radiated noise [7]. As the signals are severely corrupted by the inevitable environmental noise and transient interference after long-range transmission, feature extraction and noise mitigation of ship-radiated noise are an intricate and challenging task [3]. The signal model of ship-radiated noise can be expressed as follows [8]:

(1) x(t)=s(t)+n(t)

where x(t) is the raw signal collected by the hydrophone, s(t) is the clean signal of ship-radiated noise, and n(t) denotes complex environmental noise.

The techniques of feature extraction and noise mitigation are extensively applied to underwater targets such as underwater acoustic signal detection, sea-bottom exploration and marine biological monitoring [8] etc. Thus, the topic of advanced feature extraction and noise mitigation methods attracts extensive attention. In the past two decades, the feature extraction of ship-radiated noise has been extensively explored by developing the advanced techniques of underwater signal processing [9]. The oscillation nature, duffing oscillator [10] and stochastic resonance theory [11] are utilized to detect the line-spectrum of ship-radiated noise. According to their non-stationary nature, emerged time-frequency analysis techniques are much more suitable for non-stationary signals for combining the advantages of methods that provide the non-stationary information in the time domain and frequency domain, such as the short-time Fourier transform (STFT) [12,13], wavelet transform (WT) [7,14] and the Hilbert–Huang transform (HHT) [15,16]. Taking into consideration the non-linear nature of ship-radiated noise, numerous methods are employed for non-linear feature extraction, including phase space reconstruction [17,18], fractal-based approaches [19,20] and complexity measures [21], etc. Taking the non-stationary and non-linear features into account simultaneously, some effective algorithms were developed to accurately capture embedded non-linear and non-stationary information. For example, Fei Bao analyzed ship-radiated noise in the subspace of intrinsic mode functions in [22] that were obtained by empirical mode decomposition [23], because the extraction of non-linear features becomes much more feasible by the non-linear analysis of individual decomposed components. In [24], manifold learning using auditory model features was adopted to obtain more effective features of ship-radiated noise signals. Manifold learning is widely applied to non-linear feature extraction of diverse applications. It can visualize a low-dimensional non-linear signature hidden in high-dimensional data-processing by methods incorporating principal component analysis (PCA) [25], isometric feature mapping (IsoMap) [26], locally linear embedding (LLE) [27] and local tangent space alignment (LTSA) [28] etc. The determined information can be reserved well by manifold learning for its inherent manifold signature; however, the random noise will be eliminated as it does not have a stable skeleton form. Therefore, manifold learning also demonstrates a good noise mitigation performance. Recently, time-frequency manifold (TFM) [29] based on time-frequency distribution (TFD) and manifold learning has been proposed to extract the non-stationary and non-linear feature, and this reduces the noise that corrupts the objective signal. Noise mitigation can be classified into two categories: the filter-based and the wavelet decomposition-based strategies. The main principle of filter-based denoising algorithms is to seek out the appropriate center frequency and bandwidth, in order to preserve the narrowband signal component and discard the noise component from in-band noise. The theoretical basis of the wavelet decomposition algorithms is the idea of multi-resolution analysis [30]. Motivated by the oscillatory nature and denoising issue, resonance-based sparse signal decomposition (RSSD) [31] was proposed to extract the oscillatory signature and condense the noise. The merits of RSSD are as follows: (1) in-band noise can be removed by RSSD; (2) the oscillatory signature can be captured from a signal with severe noise corruption; (3) the prior information of the objective signals is not required.

Due to the generating mechanism of ship-radiated noise and the effect of underwater acoustic channels, a signal of ship-radiated noise has the characteristics of oscillation, non-stationary and non-linear. By considering these three characteristics simultaneously, we propose a new technique for extracting the time-frequency features of ship-radiated noise called resonance-based time-frequency manifold (RTFM). However, TFM does not provide any approach for the oscillatory nature of the vibrational signals. The main contributions are as follows: (a) the oscillatory information contained in the high-resonance component is extracted by the RSSD algorithm, which can facilitate the noise mitigation compared to the conventional TFM method; (b) the proposed algorithm is validated based on the real experimental datasets. Section 2 introduces the detail of the proposed method. Section 3 is devoted to the results and analysis of the real audio recording of ship-radiated noise. Finally, the conclusion is presented in Section 4.

## 2. Methodology

**Figure 1:**
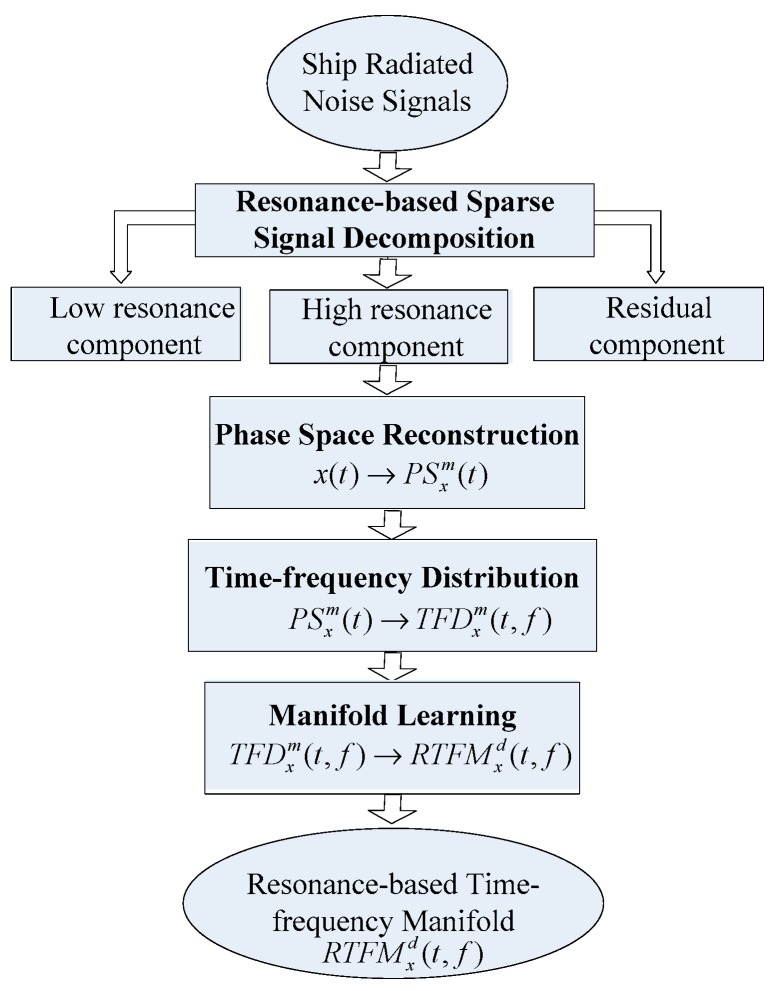
Flow chart of the resonance-based time-frequency manifold (RTFM) algorithm.

In this paper, the aim of the proposed method is to capture an effective and robust time-frequency signature of ship-radiated noise under severe noise corruption that reveals the characteristics of oscillation, non-stationary and non-linear simultaneously. To comprehend our method, four techniques are integrated as RTFM, which consists of RSSD (the link of the RSSD toolbox: http://eeweb.poly.edu/iselesni/TQWT/index.html), phase space reconstruction (PSR) (the link of the PSR codes: http://cn.mathworks.com/matlabcentral/fileexchange/54693-phase-space-reconstruction), TFD and manifold learning, as shown in Figure 1. The main principle of the RTFM is as follows. First, RSSD is employed to extract a high-oscillatory signal which represents the periodic oscillatory component hidden in the ship-radiated noise signal, and to purge low-oscillatory signal and residual signal which denote the transient pulse signal and white Gaussian noise, respectively. Second, the PSR method is used to convert the 1-D high-oscillatory signal to the multidimensional signals in the high-dimensional phase space. Third, the TFDs using a STFT spectrogram is produced in the high-dimensional phase space. Finally, manifold learning is performed on the TFDs to generate the RTFM signature which is the intrinsic non-linear feature embedded in the original signal. To improve the performance of the final RTFM signature, a synthetic TFM signature is produced by proportionally overlapping the top two RTFMs together. The RTFM is fit for extracting the inherent features hidden in the original signal depending on the combination of the characteristics of non-stationary, non-linear and oscillation. The effectiveness of the synthetic RTFM signature is validated by comparing it with the TFM method based on real ship-radiated noise signals acquired from the sea.

### 2.1. Resonance-Based Sparse Signal Decomposition

Resonance-based sparse signal decomposition is an effective technique for extracting the sustained oscillatory component that is concealed in a ship-radiated noise signal. RSSD aims to decompose the objected signals into high-oscillatory, low-oscillatory and residual components, where the high-oscillatory component is a signal consisting of multiple simultaneous sustained oscillations, the low-oscillatory component is a signal consisting of non-oscillatory transients, and the residual component is Gaussian white noise [31,32]. Both high- and low-oscillatory components may be either a high-frequency signal or a low-frequency signal. Meanwhile, the pulses in the high-oscillatory component are alien from those of the low-oscillatory component which are not reflected in the frequency domain, due to the degree of their oscillations. The high-oscillatory and low-oscillatory components cannot be extracted by frequency-based methods. Therefore, RSSD is a non-linear signal-decomposition algorithm based on the oscillatory behavior of the signals, rather than frequency or scale. Note that in-band and out-band noise can be removed by the RSSD algorithm, but the filter-based method has no ability to reduce in-band noise. A tunable Q-factor wavelet transform (TQWT) [31] and morphological component analysis (MCA) [33,34] are adopted in the RSSD algorithm. TQWT is applied to obtain the basic functions of high-Q and low-Q wavelet transforms and obtain the corresponding wavelet coefficients for signal decomposition. MCA is a general method for signal decomposition based on sparse representation, which is utilized to decompose the signal into high-oscillatory, low- oscillatory and residual components.

#### 2.1.1. Q-factor and Signal Oscillatory Behavior

The quantity of the quality factor (Q-factor) reflects the oscillatory intensity of one signal. The Q-factor is defined as follows [35]:

(2) $$Q=f_{c}/BW$$

where $f_{c}$ is the center frequency, and BW is the bandwidth. When the input signal has the sampling rate $f_{s}$, the center frequency $f_{c}$ of the level j is given by [36]:

(3) $$f_{c}=\alpha^{j}\frac{2-\beta }{4\alpha }f_{s}$$

and the bandwidth BW is expressed as [36]:

(4) $$BW=\frac{1}{2}\beta \alpha^{j-1}\pi$$

where α and β are the scaling parameters of low-pass and high-pass scaling, respectively. According to (2)–(4), the Q-factor can be derived in the form of α and β as below [32]:

(5) $$Q=\frac{2-\beta }{\beta }$$

**Figure 2:**
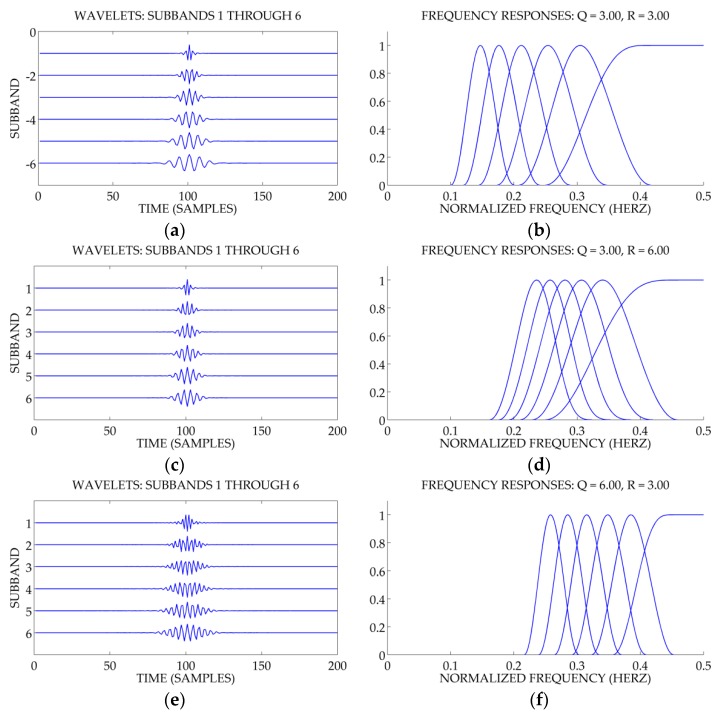
Wavelet waveforms and the corresponding spectra of frequency response under the different parameters. (**a**) waveform with Q=3,r=3. (**b**) Spectrum with Q=3,r=3. (**c**) waveform with Q=3,r=6. (**d**) Spectrum with Q=3,r=6. (**e**) waveform with Q=6,r=3. (**f**) Spectrum with Q=6,r=3.

It is obvious from Figure 2 that a signal with a higher Q-factor reveals a higher oscillatory intensity in the time-domain and, at the same time, better frequency concentration, and vice versa [36]. The suitable selection of Q-factor values for the wavelet basis functions is vital to the effective extraction of the oscillatory information that is embedded in ship-radiated noise signals. When the analyzed signal is comprised of more oscillatory components, the higher Q-factor values of the wavelet basis function should be chosen and vice versa. Compared with the frequency-based filtering methods, resonance-based methods have the overwhelming superiority that the Q-factor can be utilized to separate the signals with distinct oscillatory behaviors, even when they are distributed at the same frequencies [35].

#### 2.1.2. Tunable Q-Factor Wavelet Transform

**Figure 3:**
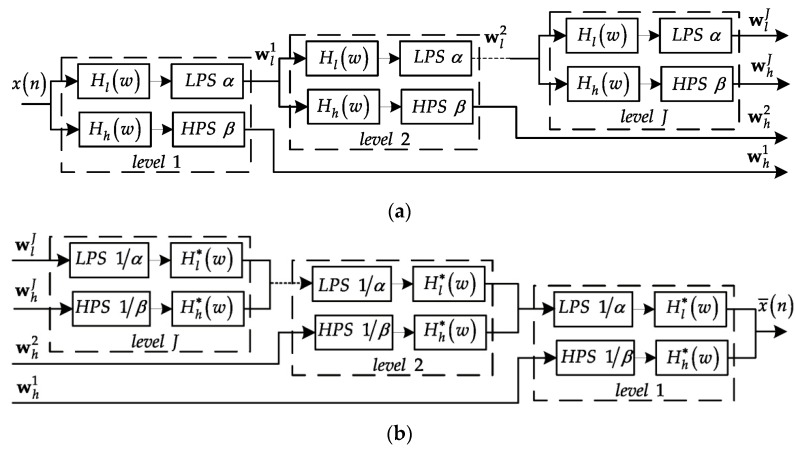
The filter banks for the tunable Q-factor wavelet transform (TQWT). (**a**) The analysis filter banks. (**b**) The synthetic filter banks.

Essentially, the tunable Q-factor wavelet transform is a discrete-time wavelet transform (DWT) [37] with adjustable constant dual Q-factors, over-complete bases and a perfect reconstruction property [32]. The flexibility of the TQWT is such that the Q-factors of DWT can be adjusted due to the oscillatory behavior of the observed signal. The frame of the TQWT is due to the discrete dyadic DWT which employs the analysis and synthesis filter banks with two channels and real-valued scaling parameters [32], as exhibited in Figure 3. For each level, two channels are made up of a high-pass filter $H_{h}\left(w\right)$ and a low-pass filter $H_{l}\left(w\right)$, where $H_{h}\left(w\right)$ and $H_{l}\left(w\right)$ are constructed as below [32]:

(6) $$H_{h}\left(w\right)=\{\begin{array}{cc} 0 & \left|w\right|\leq \left(1-\beta \right)\pi \\ \mu \left(\frac{\alpha \pi -w}{\alpha +\beta -1}\right) & \left(1-\beta \right)\pi \leq w\leq \alpha \pi \\ 1 & \alpha \pi \leq \left|w\right|<\pi \end{array}$$

(7) $$H_{l}\left(w\right)=\{\begin{array}{cc} 1 & \left|w\right|\leq \left(1-\beta \right)\pi \\ \mu \left(\frac{w+\left(\beta -1\right)\pi }{\alpha +\beta -1}\right) & \left(1-\beta \right)\pi \leq w\leq \alpha \pi \\ 0 & \alpha \pi \leq \left|w\right|<\pi \end{array}$$

where 0<β≤1, 0<α<1, and $\mu \left(w\right)=0.5(1+\cos w)\sqrt{2-\cos w}$. Then, the outputs of the filters are further handled by low-pass and high-pass scaling, where low-pass scaling with 0<α<1 is defined as [32]:

(8) Y(w)=X(αw), |w|≤π

and high-pass scaling with 0<β≤1 is expressed as [32]:

(9) $$Y\left(w\right)=\{\begin{array}{cc} X\left(\beta w+\left(1-\beta \right)\pi \right), & 0<w<\pi \\ X\left(\beta w+\left(1-\beta \right)\pi \right), & -\pi <w<0 \end{array}$$

The TQWT algorithm is executed by using the two channel filter banks on its low-pass channel iteratively and, then, further processed by the low-pass and high-pass scaling. Meanwhile, the optimal over-complete bases can be built by the selection of the oversampling rate r to attain the optimal sparse signal representation [38,39].

The most important parameters of the TQWT are the quality factor Q, the oversampling rate r and the level J. Q has been defined in Equation (5). The relationship of r, α and β can be described as follows [32]:

(10) $$r=\frac{\beta }{\alpha +1}$$

and the maximum number $J_{\max}$ of levels must be satisfied by the condition as below [32]:

(11) $$J_{\max}=\left|\frac{\log\left(\beta N/8\right)}{\log\left(1/\alpha \right)}\right|$$

where N is the length of the input signal. According to Equations (5), (10) and (11), it is obvious that the desired values of Q, r and J can be calculated by selecting the appropriate α and β. Note that the selection of parameters α and β for the objected signal is not affected by the variation of signal-to-noise ratio (SNR), as the RSSD algorithm is based on the inherent oscillatory behavior of the signals.

The selection of Q, r and J must abide by the following criteria [31,32,33,34,35,36,38]. Firstly, the number of the oscillatory wavelet. A higher value of the high Q-factor generates more oscillatory wavelets. Setting Q=1 leads to a wavelet transform for which the wavelet is similar to the second derivate of a Gaussian. Therefore, Q=1 is fit for the low Q-factor. Secondly, the assigned value of the oversampling rate r must be strictly greater than 1 and r≥3 is generally recommended to avoid the following issue. When two-channel filter banks are iterated on its low-pass output and calculated infinitely to perform a wavelet transform, the wavelet transform is oversampled by r. If r edges to unity, the transition bands of $H_{l}\left(w\right)$ and $H_{h}\left(w\right)$ will be relatively narrow and the time-domain response will not be well localized. If Figure 2b,d are compared, altering r does not change the shape of the frequency response. However, when Q is invariable, increasing r leads to aggravation of the overlap of adjacent frequency response. Finally, the level J should be set as large as possible in order to make wavelets cover frequency band maximally and get the over-complete bases, although the bigger J leads to higher computational complexity.

In essence, the TQWT method is a constant Q-factor wavelet transform. We need to set $Q_{h}$, $r_{h}$ and $J_{h}$ for a high-oscillatory channel and $Q_{l}$, $r_{l}$ and $J_{l}$ for a low-oscillatory channel manually. The selection guide of the above parameters is given in Table 1 [38].

#### 2.1.3. Morphological Component Analysis

Morphological component analysis has been developed to separate different morphological features based on sparse representation [33]. The oscillatory and non-oscillatory components which are hidden in ship-radiated noise are taken for the disparate morphological features, so MCA can be applied to isolate and extract the oscillatory component. The aim of MCA is to construct the optimal sparse representation of high-resonance and low-resonance components, then separate these two components. Suppose a ship-radiated signal $x=x_{h}+x_{l}+n$, $x,x_{h},x_{l},n\in {ℜ}^{N}$, where $x_{h}$, $x_{l}$ and n represent the high-oscillatory component, low-oscillatory component and residual component, respectively. Assume that $x_{h}$ and $x_{l}$ can be represented sparsely in basis $\Psi_{h}$ and $\Psi_{l}$. The aim of MCA is to estimate $x_{h}$ and $x_{l}$ individually which can be determined by minimizing the objective function as follows [31,34]:

(12) $$J\left(w_{l},w_{h}\right)={\left\|x-\Psi_{l}w_{l}-\Psi_{h}w_{h}\right\|}_{2}^{2}+\sum_{j=1}^{J_{1}+1}\lambda_{l,j}{\left\|w_{l}^{j}\right\|}_{1}+\sum_{j=1}^{J_{2}+1}\lambda_{h,j}{\left\|w_{h}^{j}\right\|}_{1}$$

where $w_{l}^{j}$ and $w_{h}^{j}$ are the wavelet coefficients of low-oscillatory and high-oscillatory components for each level, respectively, $J_{1}$ and $J_{2}$ are the levels of low-oscillatory and high-oscillatory components, $\lambda_{l,j}$ and $\lambda_{h,j}$ are the regularization parameters which are the metric of the sparse representation of $w_{l}$ and $w_{h}$. For the level j, the values of $\lambda_{l,j}$ and $\lambda_{h,j}$ are determined by the proportion of the energy of $\Psi_{l,j}$ and $\Psi_{h,j}$, expressed as [39]:

(13) $$\lambda_{l,j}=c_{l,j}{\left\|\Psi_{l,j}\right\|}_{2},\;\lambda_{h,j}=c_{h,j}{\left\|\Psi_{l,j}\right\|}_{2}$$

where $c_{l,j}+c_{h,j}=1$, $c_{l,j}$ and $c_{h,j}$ are the proportion parameters of the energy distribution between low-oscillatory and high-oscillatory components. Note that when $\lambda_{h}$ is constant, increasing $\lambda_{l}$ will enhance the energy of $x_{h}$ and impair the energy of $x_{l}$, and vice versa. Furthermore, augmenting both of $\lambda_{h}$ and $\lambda_{l}$ will strengthen the energy of the residual and weaken that of the oscillatory components. Therefore, we set $c_{l,j}=c_{h,j}=0.5$ in this paper for all levels j to balance the energy distribution of these two components.

Then $w_{l}^{*}$ and $w_{h}^{*}$ are obtained by using the split augmented Lagrangian shrinkage algorithm (SALSA) [31], to minimize the objective function in Equation (12). For details of the SALSA algorithm refer to [31]. MCA provides the estimation of high- and low-oscillatory components as follows [31]:

(14) $${\hat{x}}_{h}=\Psi_{h}w_{h}^{*},\;{\hat{x}}_{l}=\Psi_{l}w_{l}^{*}$$

where ${\hat{x}}_{h}$ is high-oscillatory signal and ${\hat{x}}_{l}$ is low-oscillatory signal.

In conclusion, the RSSD algorithm can be summarized in Algorithm 1. The high-oscillatory component ${\hat{x}}_{h}$ is extracted as the input signal of the PSR method, which is used to transfer one-dimensional objected signal to high-dimensional phase space.
**Algorithm 1.** Resonance-based sparse signal decomposition (RSSD).**Input:** The raw signal of ship radiated noise x(t). **Output:** The high-oscillatory signal ${\hat{x}}_{h}$ and the low-oscillatory signal ${\hat{x}}_{l}$.Initialize: **Set the suitable α and β.****1: Calculate $Q_{h}$, $r_{h}$ and $J_{h}$ for high-oscillatory channel and $Q_{l}$, $r_{l}$ and $J_{l}$ for low-oscillatory channel;****2: Construct the wavelet bases $\Psi_{h}$ and $\Psi_{l}$ by TQWT based on the above selected parameters;****3: Choose the benefitting weight parameters $\lambda_{l,j}$ and $\lambda_{h,j}$ at each level according to Equation (13) and observation of noise corruption intensity;****4: Work out the objected optimization Function (12) using the SALSA method and obtain the wavelet coefficient matrices $w_{l}^{*}$ and $w_{h}^{*}$;****5: Estimate high-oscillatory and low-oscillatory components ${\hat{x}}_{h}$ and ${\hat{x}}_{l}$ according to Equation (14).**

### 2.2. Phase Space Reconstruction

Phase space reconstruction [40] is an efficient method for searching for an inherent pattern of dynamic system embedded in the time series by utilizing time-delayed versions of a time series as coordinates for the space. This algorithm aims to depict the orbit of the dynamic system in the reconstructed high-dimensional space [29]. It is the most used method of PSR is the time-delay reconstruction that provides a coordinate system in essence [41,42,43,44]. For extracting the manifold of ship-radiated noise signals, the high-dimensional phase space data is obtained by projecting a one-dimensional high-oscillatory signal ${\hat{x}}_{h}$ to the phase space by PSR. However, the estimation of the embedding dimension m does not require prior knowledge. In this paper, we used Cao’s method [45] to decide the embedding dimension m due to its superiority of robustness to noise.

Let $\left\{{\hat{x}}_{i},i=1,2,\ldots ,N\right\}$ be an observed time series with one dimension. Assume the embedding dimension is m, the jth vector in the m-dimensional phase space can be reconstructed by the following equation [44]:

(15) $$X_{j}^{m}=\left[{\hat{x}}_{j},{\hat{x}}_{j+\tau },\ldots ,{\hat{x}}_{j+\left(m-1\right)\tau }\right]$$

where ${\hat{x}}_{j}$ is the *j*th data point of the high-resonance signal $\hat{x}\left(t\right)$ and τ is the time delay. Note that the time delay τ=1 could be set by Takens’ theory [46]. In this paper, we have chosen τ=1 to obtain a high time resolution of RTFM.

In Cao’s method, the mean value $E_{1}\left(m\right)$ is defined to diagnose a false neighbor as below [45]:

(16) $$E_{1}\left(m\right)=E\left(m+1\right)/E\left(m\right)$$

where,

(17) $$E\left(m\right)=\frac{1}{N-m\tau }\sum_{i=1}^{N-\left(m-1\right)\tau }\frac{\left\|X_{j}^{m+1}-X_{\eta \left(j\right)}^{m+1}\right\|}{\left\|X_{j}^{m}-X_{\eta \left(j\right)}^{m}\right\|}$$

when m is larger than a constant value $m_{0}$, the embedding dimension $m_{0}$ is determined under the condition that $E_{1}\left(m\right)$ would become a steady value.

High-dimensional data vectors $\left\{P_{x}^{i},i=1,2,\ldots ,m\right\}$ can be constructed from a one-dimensional observed signal by PSR. A m×n matrix in the phase space can be formed from these vectors as follows [40]:

(18) $$\left[\begin{array}{c} P_{x}^{1}\\ P_{x}^{2}\\ \cdots \\ P_{x}^{m} \end{array}\right]=\left[\begin{array}{c} X_{1}^{m}\\ X_{2}^{m}\\ \cdots \\ X_{n}^{m} \end{array}\right]=\left[\begin{array}{cccc} x_{1} & x_{2} & \cdots & x_{n}\\ x_{2} & x_{3} & \cdots & x_{n+1}\\ \cdots & \cdots & \cdots & \cdots \\ x_{m} & x_{m+1} & \cdots & x_{N} \end{array}\right]$$

where n=N−m+1 and $P_{x}^{i}\in R^{1\times n},i=1,2,\ldots ,m$. The time-series vectors $\left\{P_{x}^{i},i=1,2,\ldots ,m\right\}$ can be regarded as m-dimensional signals in the phase space.

### 2.3. Time-Frequency Distribution

Time-frequency distribution is the dominant tool for providing the information of a time-frequency domain for non-stationary signals. TFD performs a mapping of the one-dimensional signal into a two-dimensional signature combining the time and frequency information. It is well known that STFT is one of earliest time-frequency analysis method and still one of most widely used [47,48]. The time-frequency distribution using STFT [46] is called the spectrogram. The spectrogram is a positive and real-valued distribution which exposes a synthetic structure of ship-radiated noise. In this paper, we adopted the spectrogram to depict the TFD of constructed signals in the phase space. TFD is formulated as below [48]:

(19) $$TFD_{x}^{m}(t,f)={\left|\int_{-\infty }^{+\infty }P_{x}^{m}\left(\tau \right)h^{*}\left(t-\tau \right)e^{-i2\pi f\tau }d\tau \right|}^{2}$$

where h(t) is a STFT window which is centralized at t=0 and f=0 and $h^{*}\left(t\right)$ is the complex conjugate of h(t). When the spectrogram is employed as high-dimensional vector $\left\{P_{x}^{i}|i=1,2,\ldots ,m\right\}$, each 1×n vector $P_{x}^{i}$ is mapped to the $n^{*}\times L$ constructed TFD in the phase space where $n^{*}$ is smaller than n. For m-dimensional phase space, a 3-D matrix with the size of $m\times n^{*}\times L$ is formed by all the TFDs.

### 2.4. Manifold Learning

Non-linear manifold learning is an emerging and effective method of dimension reduction to visualize the low-dimensional non-linear manifold features from the unorganized high-dimensional data. In this paper, we conducted LTSA (the link of the LTSA code: https://github.com/gionuno/local_tangent_space_alignment) [28] on 3-D TFDs with the size of $m\times n^{*}\times L$ in high-dimensional phase space to realize dimension reduction and non-linear manifold extraction. In mathematics, we assume that a d-dimension manifold hidden in an m-dimensional phase space (d<m) can be formulated as follows [28]:

(20) $$f:\;\alpha_{i}\in {ℜ}^{d}\to \varphi_{i}\in {ℜ}^{m}$$

where $\varphi_{i}$ is the resolution data of i-th TFD in the m-dimensional phase space and $\alpha_{i}$ is the low-dimensional reconstructed feature vector.

The main principle of the LTSA algorithm is described as follows [28,49]: firstly, LTSA indicates the local geometry of the manifold utilizing tangent spaces which are learned through fitting an affine subspace in a neighborhood of each data point. Secondly, LTSA aligns these tangent spaces to acquire the global coordinates of each data sample in regard to the underlying manifold by a partial eigen decomposition of the neighborhood connection matrix [50,51]. The determined non-linear information is well reserved by the LTSA algorithm on account of its intrinsic manifold, whereas the random information (e.g., the noise) is abandoned because the inherent solid structure does not exist in the noise [52]. We have known that the size of the 3-D TFDs matrix is $m\times n^{*}\times L$. In order to adapt the input of the LTSA algorithm, we need to transform 3-D matrix to 2-D by concatenating column by column for each 2-D TFD matrix. After data processing, then LTSA is implemented on the 2-D recombinant TFD matrix with the size of $m\times \left(n^{*}\times L\right)$.

We assume a data set $\Phi =\left[\varphi_{1},\ldots ,\varphi_{\left(n^{*}\times L\right)}\right]\in {ℜ}^{m\times \left(n^{*}\times L\right)}$ with $\varphi_{i}\in {ℜ}^{m}$, which denotes the TFD pixels in m-dimensional phase space. The d-dimensional coordinates $\Lambda \in R^{d\times \left(n^{*}\times L\right)}$ are generated by the LTSA method to construct the manifold from a data of local nearest neighbors. The LTSA method is performed in the following steps [28,29]:

Step 1: Determining local neighborhood: the set of k neighbors $\Phi_{i}=\left[\varphi_{i_{1}},\ldots ,\varphi_{i_{k}}\right]$ for each data point $\varphi_{i}$ is selected by the Euclidean distance $\Delta ={\left\|\varphi_{i}-\varphi_{i_{j}}\right\|}_{2}^{2},j=1,2,\ldots ,k$.

Step 2: Local linear fitting: the basic model of the LTSA algorithm is to discover the optimal d-dimensional affine subspace approximation for the set of k neighbors $\Phi_{i}$ by the following optimization equation [28]:

(21) $$\underset{\varphi ,\Theta ,Q}{\min}\sum_{j=1}^{k}{\left\|\varphi_{i_{j}}-\left(\varphi +\Omega \theta_{j}\right)\right\|}_{2}^{2}=\underset{\varphi ,\Theta ,Q}{\min}{\left\|\Phi_{i}-\left(xe^{T}+\Omega \Theta \right)\right\|}_{2}^{2}$$

where Ω is an orthonormal basis matrix with d columns and Θ is the local coordinates series. The optimal φ is obtained by ${\bar{\varphi }}_{i}$ which is the mean of all the $\varphi_{i_{j}}$ where j=1,2,…,k, the optimal Ω is obtained by $\Omega_{i}$ that can be taken as the d left singular vectors of $\Phi_{i}\left(I-ee^{T}/k\right)$ corresponding to its d largest singular values by singular value decomposition (SVD) [53], i.e., $\Phi_{i}\left(I-ee^{T}/k\right)=\Omega_{d}\Sigma_{d}V_{d}^{T}$, and Θ is obtained by $\Theta_{i}=\left[\theta_{1}^{\left(i\right)},\ldots ,\theta_{k}^{\left(i\right)}\right]$ where $\theta_{j}^{\left(k\right)}=\Omega_{i}^{T}\left(\varphi_{i_{j}}-{\bar{\varphi }}_{i}\right)$. Therefore, we can gain $\left(n^{*}\times L\right)$ local coordinates $\Theta_{i}=\left[\theta_{1}^{\left(i\right)},\ldots ,\theta_{k}^{\left(i\right)}\right]$, $i=1,\ldots ,n^{*}\times L$.

Step 3: Constructing alignment matrix: for aligning $\left(n^{*}\times L\right)$ local coordinates $\Theta_{i}=\left[\theta_{1}^{\left(i\right)},\ldots ,\theta_{k}^{\left(i\right)}\right]$, $i=1,\ldots ,n^{*}\times L$ to get global coordinates $G_{i}=\left[g_{i_{1}},\ldots ,g_{i_{k}}\right]$, $i=1,\ldots ,n^{*}\times L$, the objective is to strive for seeking out $g_{i}$ and the optimal alignment matrix $L_{i}$ in order to minimize the reconstruction errors $E_{i}=G_{i}\left(I-ee^{T}/k\right)-L_{i}\theta_{i}$ as follows [28]:

(22) $$\min{\sum_{i}\left\|E_{i}\right\|}^{2}\equiv {\sum_{i}\left\|G_{i}\left(I-ee^{T}/k\right)-L_{i}\theta_{i}\right\|}^{2}$$

where e is an column vector of all ones. For the fixed $G_{i}$, the optimal $L_{i}$ is obviously given by $L_{i}=G_{i}\left(I-ee^{T}/k\right)\Theta_{i}^{†}=G_{i}\Theta_{i}^{†}$, where † represents the Moore–Penrose generalized inverse. Then, we need to find G to minimize the overall reconstruction error defined as follows [28]:

(23) $$\sum_{i}{\left\|E\right\|}_{F}^{2}=\sum_{i}{\left\|GS_{i}\Lambda_{i}\right\|}_{F}^{2}={\left\|GS\Lambda \right\|}_{F}^{2}$$

where $G=\left[g_{1},\ldots ,g_{n^{*}\times L}\right]$ and $S=\left[S_{i_{1}},\ldots ,S_{n^{*}\times L}\right]$. here $S_{i}$ is the 0−1 selection matrix such that $GS_{i}=G_{i}$ and $\Lambda =diag\left(\Lambda_{1},\ldots ,\Lambda_{\left(n^{*}\times L\right)}\right)$ with $\Lambda_{i}=\left(I-ee^{T}/k\right)\left(I-\Theta_{i}^{†}\Theta_{i}\right)$. To uniquely determine G, we impose the constraints $GG^{T}=I_{d}$. So the alignment matrix can be constructed as $B\equiv S\Lambda \Lambda^{T}S^{T}$.

Step 4: Aligning global coordinates: first, compute the d+1 minimum eigenvectors of B.

Second, obtain the eigenvector matrix $\left[u_{2},\ldots ,u_{d+1}\right]$ corresponding to the 2nd to d+1 minimum eigenvalues; Finally, set the global coordinates $G={\left[u_{2},\ldots ,u_{d+1}\right]}^{T}$.

The global coordinates of the low-dimensional RTFMs are equal to $G={\left[u_{2},\ldots ,u_{d+1}\right]}^{T}$. The 3-DRTFM matrix can be recombined with the size of $d\times n^{*}\times L$ where d≪m. We denote this 3-D RTFM matrix as $RTFM_{x}^{d}\left(t,f\right)$.

## 3. Results and Discussion

In this paper, we used a real audio recording of ship-radiated noise which was recorded by an underwater hydrophone in the shallow sea located on the west coast of Taiwan Strait and named as ship A. The real observed signal, which is a time series with the data points N=2048, is acquired under the condition that the sampling frequency is 10 KHz, the depth of the underwater hydrophone is 25 m, and the distance between the objectd ship and the hydrophone is about 1 km.

**Figure 4:**
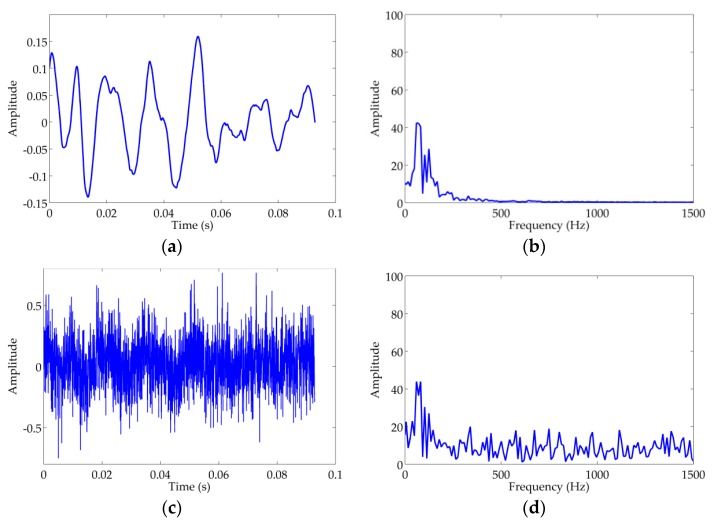
Waveform and spectrum of the recording audio for ship radiated noise. (**a**) The clean ship-radiated noise. (**b**) Spectrum of the clean ship-radiated noise. (**c**) Ship-radiated noise (SNR=−10 dB). (**d**) The spectrum of signal (SNR=−10 dB).

In this paper, the audio recordings of ship-radiated noise are regarded as the clean signals, because the raw signal is less corrupted by the inevitable noise shown in Figure 4a. It is obvious that the clean ship-radiated noise is a periodic oscillatory signal in a time domain and the main spectral energy concentrates below 200 Hz, as demonstrated in Figure 4a,b. Thus, in essence ship-radiated noise is a low-frequency periodic oscillatory signal. It is necessary to verify the performance of RTFM under severe noise pollution and ship-radiated noise with −10 dB adopted as the observed signal, as shown in Figure 4c,d. For the real oceanic experiment, we apply the RTFM algorithm to extract the 2-D effective manifold signature of ship-radiated noise for visualizing the oscillatory, non-stationary and non-linear features and eliminating the noise and interference. In this section, we demonstrate the experimental results of RTFM signatures and synthetic RTFM signatures under severe noise corruption. Meanwhile, we verify the effectiveness of the RTFM approach by comparing it with TFM. Note that, all of the case studies were done against an existing database of real audio recordings of ship-radiated noise.

### 3.1. Results and Analysis of Resonance-Based Time-Frequency Manifold (RTFM)

**Figure 5:**
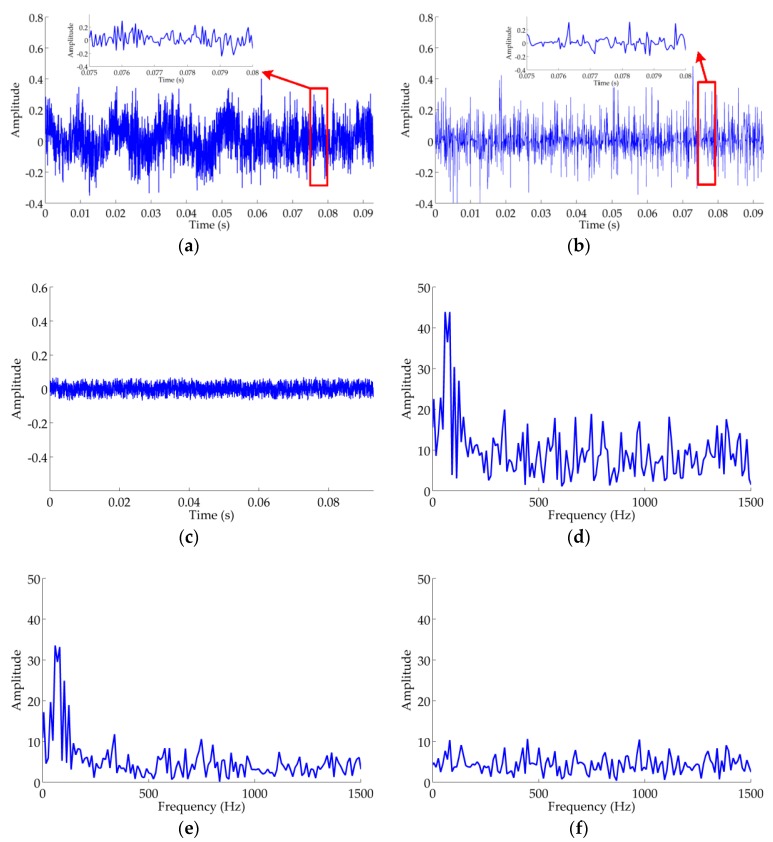
Waveform and spectra of ship-radiated noise using RSSD algorithm. (**a**) Waveform of high-resonance component. (**b**) Waveform of low-resonance component. (**c**) Waveform of residual component. (**d**) Spectrum of the raw signal. (**e**) Spectrum of high-resonance component. (**f**) Spectrum of low-resonance component.

According to the process of the RTFM algorithm, firstly, we conducted the RSSD algorithm on the observed signal to extract the high-resonance component. We set the scaling parameters α = 0.867 and β = 0.4 for high-Q TQWT, hence we obtained $Q_{1}=4$, $r_{1}=3$, $J_{1,\max}\approx 32.43$ according to Equations (5)–(7), respectively, then chose $J_{1}=32$. Likewise, α = 0.667 and β = 1 were chosen for low-Q TQWT, then we gained $Q_{2}=1$, $r_{2}=3$, $J_{2}=3$. RSSD decomposes the observed signal into high-oscillatory, low-oscillatory and residual components, which are shown in Figure 5a–c, respectively. In the two sub-figures of Figure 5a,b, we zoom locally in on the time interval [0.75:0.8] to observe the oscillatory and pulse components. It is obvious from Figure 5d–f that the spectrum of the high-oscillatory component is similar to the original signal and the inherent oscillatory information of ship-radiated noise is kept well in the high-oscillatory component. Therefore, we extract the high-oscillatory component, and meanwhile eliminate low-oscillatory and residual components which denote transient interference and white Gaussian noise, respectively.

**Figure 6:**
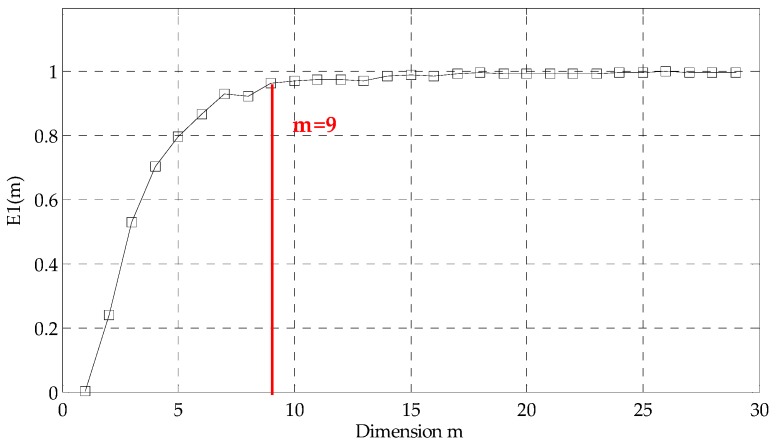
The selection of the embedding dimension.

**Figure 7:**
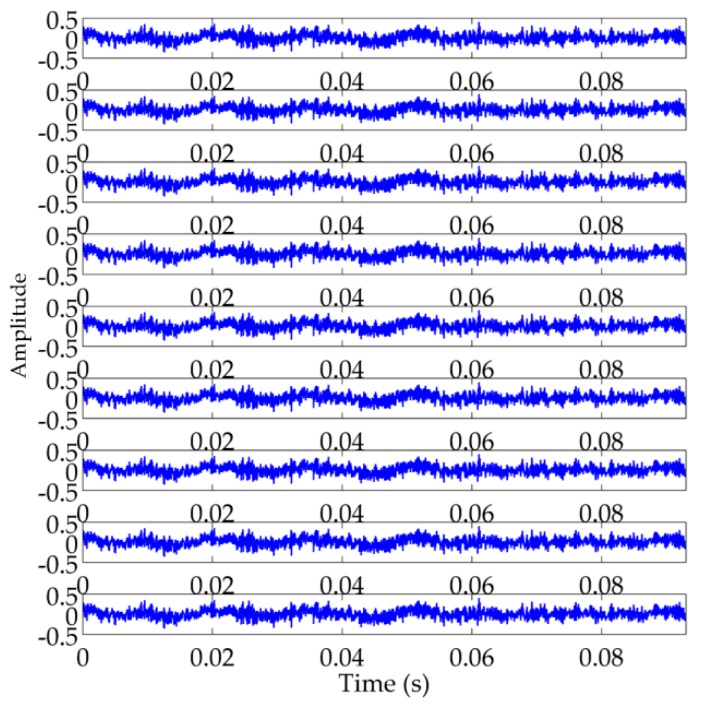
The waveforms of the constructed signals with one dimension in the phase space using phase space reconstruction (PSR).

Secondly, we employed Cao’s method to ascertain the embedding dimension and conduct PSR on high-oscillatory component extracted by RSSD to convert 1-D high-oscillatory signal to multidimensional signal in the high-dimensional phase space. For obtaining a high time resolution of RTFM, we set τ=1 and calculate $E_{1}\left(m\right)$ which is exhibited in Figure 6. It is evident that the value of $E_{1}\left(m\right)$ ceases to change basically after m=9. Therefore, the embedding dimension is selected as 9 according to Cao’s method. After determining the embedding dimension m=9, the 9-dimensional signals are constructed in the phase space in Figure 7.

**Figure 8:**
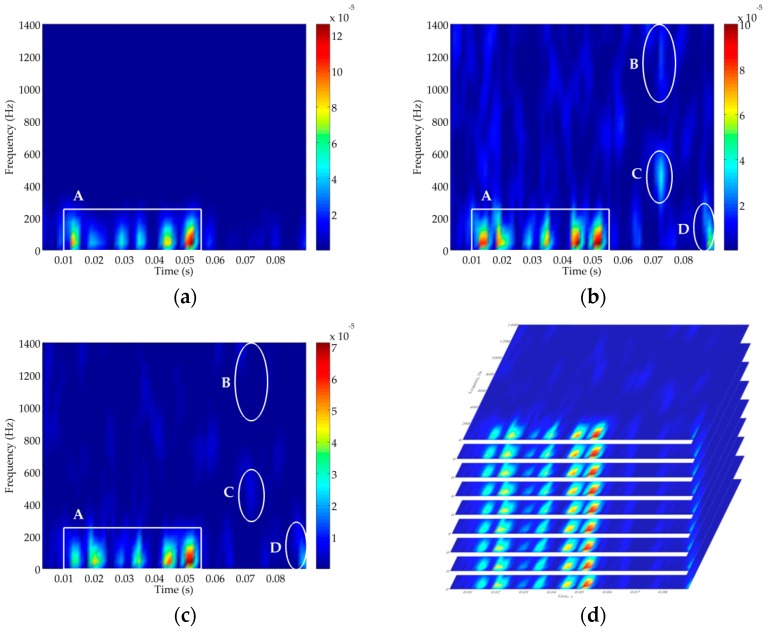
Time-frequency distribution (TFD) of ship A. (**a**) The clean ship-radiated noise. (**b**) Ship-radiated noise (SNR=−10 dB). (**c**) The high-resonance component extracted by RSSD. (**d**) m TFDs in high-dimensional phase space.

Thirdly, the TFD technique is used on the above constructed high-dimensional signals to reveal non-stationary information in the phase space. As shown in Figure 8a, it is evident that the TFD of the raw signal has high signal strength and low noise interference. Therefore, we assume that the original recording acquired by the hydrophone is the clean signal without the noise. In Figure 8b, the time-frequency resolution of the TFD is severely corrupted by noise interference for the original signal with −10 dB. Compared with the TFD of the clean signal in Figure 8a, we depict the signal and noise components where the rectangle A is the signal component and the three ellipses B, C and D are the noise components which are the most severe places of noise corruption in the TFD. Due to the effect of noise suppression using the RSSD algorithm, the TFD of high-oscillatory component is demonstrated in Figure 8c where it can be found that RSSD eliminates the noise at all scales to some extent, but the noise cannot be completely abolished especially in the ellipse D. As the time-delay operation of the PSR method just provides a time-delayed representation for each TFD in phase space, noise corruption also occurs in the phase space as illustrated in Figure 8d.

**Figure 9:**
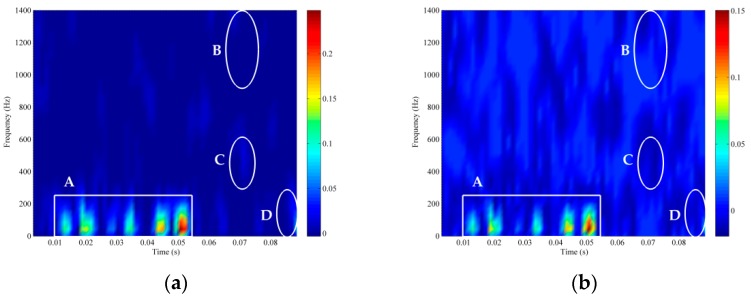
First two RTFMs signature of ship radiated noise of ship A. (**a**) 1st RTFM signature. (**b**) 2nd RTFM signature.

**Figure 10:**
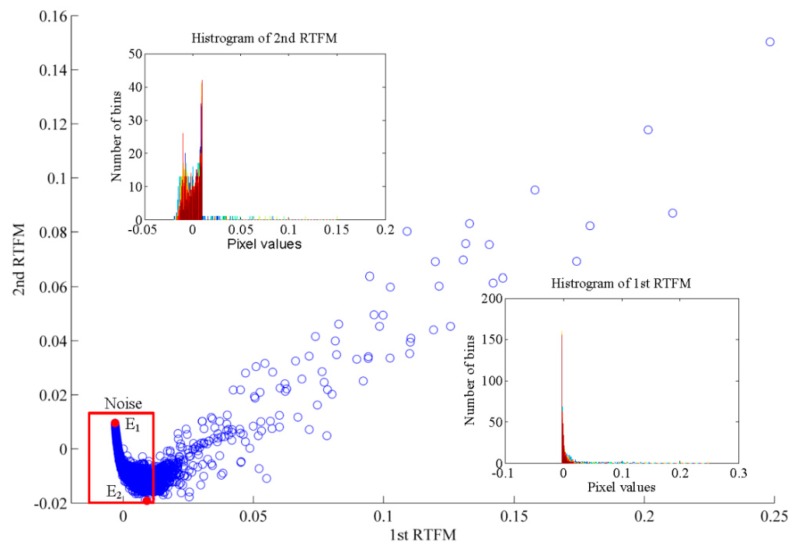
Scatter plot of first two RTFMs signatures produced by LTSA algorithm.

Finally, the LTSA algorithm is applied to the TFDs to extract the intrinsic non-linear manifold signature and eliminate the noise. The first two RTFM signatures are acquired by the LTSA method, as indicated in Figure 9, where rectangle A is the objected signal component and ellipses B, C and D are the noise components. As illustrated in Figure 9a, it is quite visible that the bigger resolution values of both the signal and noise components are reserved after the process of dimension reduction and all of their values are positive. In Figure 9b, we can find that the resolution values of the signal component remain positive; nevertheless, the resolution values of the noise components in ellipses B, C and D mostly remain negative. The two patterns can be distinctly projected into the scatter plot of these two RTFM signatures, as indicated in Figure 10, where each element signifies one pixel value in RTFM signature. In the two sub-figures, the facts are proved once again that the pixel values of the first RTFM signature are positive and the values of the noise components which exist in the second RTFM signature are negative. We can find that the amplitude is the pixel values of the first RTFM, and is a monotonic increase, seen from the *x*-axis. Different to the first RTFM, the second RTFM is a convex function in the rectangular area. The signal and noise parts are separated by the peak of the convex function where the rectangle region is noise components. Thus, we can extract the skeleton pixels from the noise by combining the first two RTFM signatures.

### 3.2. Results and Analysis of Synthetic RTFM Signatures

The idea of combining the first two RTFM signatures, which is referred to as the synthetic RTFM signature, can effectively remove the noise components and highlight the intrinsic manifold to improve the quality of the RTFM signature. In Figure 10, it is obvious that the positive/negative values of the first RTFM correspond to the negative/positive values of the second RTFM and the zero values are close to the original point of scatter plot in the rectangle area which is the noise part. Therefore, the synthetic RTFM signature can be obtained by a straightforward proportional addition. The synthetic RTFM signature can be defined by the following equation [29]:

(24) $$RTFM\left(t,f\right)=RTFM_{1}\left(t,f\right)+\eta RTFM_{2}\left(t,f\right)$$

where η is an appropriate ratio parameter for well eliminating the noise components and RTFM(t,f), $RTFM_{1}\left(t,f\right)$ and $RTFM_{2}\left(t,f\right)$ represent the synthetic RTFM signature, the first RTFM signature and the second RTFM signature, respectively. The extreme method is used to calculate η in this paper. Two extreme points $E_{1}=\left(\xi_{RTFM1}^{1},\xi_{RTFM2}^{1}\right)$ and $E_{2}=\left(\xi_{RTFM1}^{2},\xi_{RTFM2}^{2}\right)$ are calculated in the noise part of Figure 10, respectively. Therefore, η can be given as follows [29]:

(25) $$\eta =\left|\frac{\xi_{RTFM1}^{1}-\xi_{RTFM1}^{2}}{\xi_{RTFM2}^{1}-\xi_{RTFM2}^{2}}\right|$$

Note that the positive/negative values of the first RTFM are in line with the negative/positive values of the second RTFM. In conclusion, the synthetic RTFM signature can be adopted for the time-frequency feature representation of ship-radiated noise. To validate the availability and robustness of the proposed RTFM method, case studies of ship-radiated noise signals to the proposed method were conducted in the following part.

#### 3.2.1. Case Study for the Availability of the Proposed RTFM Method

In this case, we verified the effectiveness of the proposed RTFM method between the real audio recording of ship A and B with −10 dB, where the description of ship A has given in the Section 3.1 and ship B was the acoustic signal of a large commercial ship downloaded from Discovery of Sound in the Sea (http://dosits.org/galleries/audio-gallery/anthropogenic-sounds/ship/). Ship B was acquired at approximately 20 knots of the cruising speed and 3.2 km away from the hydrophone. The fragments with 2048 samples intercepted from the recordings of both ship A and B were employed as the clean signals. White Gaussian noise with −10 dB was then added to these two clean signals to produce the objected signals.

TFM method has been applied to extract the non-stationary and nonlinear features and the fact has been verified that the performance of TFM method is better than the wavelet-packing transform (WPT)-based filtering, the EMD-based filtering, the continuous wavelet transform (CWT)-based filtering, the discrete wavelet transform (DWT)-based de-nosing method and the time-domain manifold signature method in [29]. Therefore, we select TFM method as a comparison in this paper.

**Figure 11:**
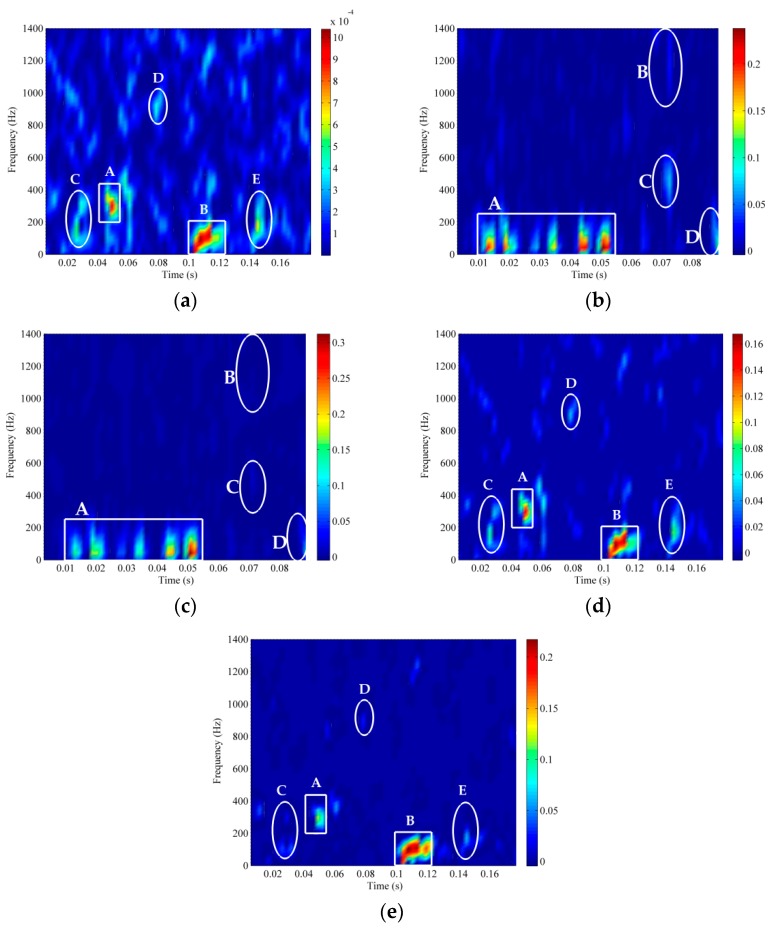
Comparison results of ship A and B (SNR=−10 dB) between the proposed RFTM and traditional methods. (**a**) The TFD of ship B. (**b**) Synthetic TFM signature of ship A. (**c**) Synthetic RTFM signature of ship A. (**d**) Synthetic TFM signature of ship B. (**e**) Synthetic RTFM signature of ship B.

The essential difference between RTFM and TFM is that the RTFM method considers the nature of oscillation, non-stationary and nonlinear simultaneously, but TFM don’t has the ability to reveal oscillatory feature. When RTFM method is operated on ship A and B, the RSSD parameters are determined artificially and the embedding dimension m is selected by Cao’s method. We set the parameters as follows: the parameters of RSSD algorithm for ship A were chosen as mentioned in Section 3.1 and for ship B the parameters were set as $Q_{1}=8$, $r_{1}=3$, $J_{1}=28$, $Q_{2}=1$, $r_{2}=3$, $J_{2}=3$ and m=6. The proposed RTFM method was applied to the ship A and B with −10 dB to extract the effective time-frequency feature signature and denoise the signature. Meanwhile, it was compared with the traditional TFM method to prove the availability and merits of the RTFM method in Figure 11. In Figure 8b and Figure 11a, both of ship A and B are contaminated by the noise, where rectangle and ellipse denote the objected signal and noise, respectively. Synthetic TFM method is the efficient method to reveal the non-stationary and nonlinear features and overall has a decent performance of noise mitigation for both ship A and B, which are exhibited in Figure 11b and Figure 10d, compared with the TFD of the original signals in Figure 8b and Figure 11a. However, some distinct noise components are still doggedly reserved in the TFM signature. Especially, the ellipses B, C and D in Figure 11b and the ellipses C and D in Figure 11d can’t be well eliminated. When we compare the RTFM signatures of ship A and B as illustrated in Figure 11c,e with their TFM signatures, it is conspicuous that the noise components are vanished in the ellipses B C and D of ship A and the ellipses C and D of ship B respectively. Although some signal components with low strength of ship A and B are slightly weakened such as rectangle A in Figure 11e, it does not affect the effective RTFM representation to a large extent. In conclusion, the performance of noise mitigation of RTFM method is much more prominent than TFM method. Meanwhile, RTFM signature is more effective than TFM signature in revealing the inherent time-frequency manifold structure related to feature extraction of ship radiated noise by carefully considering the trade-off between feature representation and noise mitigation.

#### 3.2.2. Case Study for the Robustness of the Proposed RTFM Method

**Figure 12:**
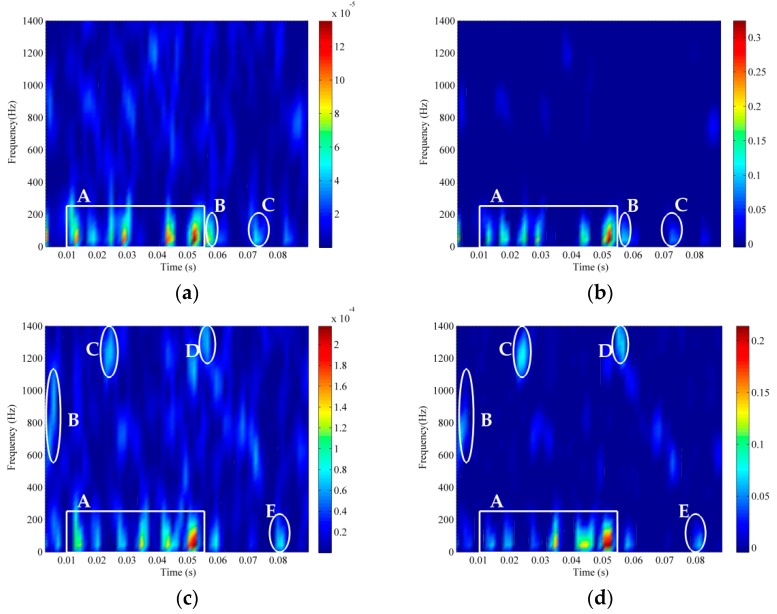
Comparison results of ship A under different SNRs using the proposed RFTM method. (**a**) The TFD of ship A (SNR=−12 dB). (**b**) Synthetic RTFM signature of ship A (SNR=−12 dB). (**c**) The TFD of ship A (SNR=−15 dB). (**d**) Synthetic TFM signature of ship A (SNR=−15 dB).

To further evaluate the robustness of the proposed method, the RTFM method was conducted on the real recording of ship A under the different SNRs which are −10 dB, −12 dB and −15 dB. In Figure 12, rectangles and ellipses also represent the signal and noise components, respectively. It is obvious that the time-frequency signatures of ship A deteriorated much more severely with the decrease of SNR, which is shown in sequence in Figure 8b and Figure 12a,c. We can find that noise components in the elliptical area as displayed in Figure 11c were perfectly removed. Figure 12b shows noise elements in the ellipses B and C are marginally retained. In Figure 12d, noise ingredients in the elliptical region have a relatively higher resolution, but the objected signal constituents in rectangle A still can be recognized readily and the RTFM method also shows a fairly good ability to eliminate noise on the whole RTFM signature. Although the resolution strength of rectangle A is somewhat impaired with the reduction of SNR, RTFM signatures also successfully recover the objected signal components from the original signal aggravated intensively by the white Gaussian noise. To balance the trade-off deliberately between feature representation and noise elimination, the effectiveness and robustness of the RTFM method can be manifested by the above experimental results. The RTFM method is practical for disparate ship-radiated noise collected from various ships and different levels of noise pollution. In conclusion, the RTFM algorithm has the case of low SNRs.

## 4. Conclusions

In order to obtain more effective feature extraction and noise mitigation for ship-radiated noise, this paper has proposed a novel RTFM signature which is comprised of RSSD, PSR, TFD and LTSA based on simultaneously considering the characteristics of oscillation, non-stationary and non-linear. The proposed RTFM signature skillfully visualizes a 2-D inherent time-frequency structure and extracts the intrinsic oscillatory, non-stationary and non-linear information embedded in ship-radiated noise. Hence, it is important to solve the difficulty of extracting the oscillatory, non-stationary and non-linear features for ship-radiated noise.

To verify the prominent effectiveness and robustness of the RTFM method, case studies were conducted on different datasets of ship-radiated noise and various degrees of noise pollution. All of the case studies are done against an existing database of real audio recordings of ship-radiated noise. Firstly, we validated the merits of the RTFM method by comparing the experimental results with the TFM method based on the objected signal of ship A with −10 dB. In this case, the experimental results indicate that RTFM has a better performance of feature representation and noise mitigation than TFM in the whole time-frequency signature. Secondly, the RTFM algorithm was applied to diverse real audio recordings of ships A and B with −10 dB, to prove further that the availability of the proposed method was useful for various ship-radiated noise signals. Finally, we conducted the RTFM method on the real audio recording of ship A under three diverse degrees of noise pollution, and the experimental results demonstrated the RTFM method has prominent robustness for different noise-pollution levels. In conclusion, the RTFM algorithm has remarkable robustness of time-frequency representation and noise suppression in the context of low SNRs.

In future, we will conduct further research on feature extraction based on the RTFM signature and image-processing techniques with the aim of recognizing ship-radiated noise signals.

## Figures and Tables

**Table 1:** The selection guides of the parameters for the TQWT algorithm [38].

Parameters	The Overlap of Frequency Response	Computational Cost	Selection Guide
Q	No direct effect	↓ if Q↑, when J=max	$Q_{l}=Q_{\min}=1$ $Q_{h}=$ oscillatory level of the signal
r	No direct effect	↑ if r↑, when J=max	r= trade-off between overlapping intensity of frequency responses and computational cost
J	↑ if r↑	↑ if J↑	$J=J_{\max}$
